# TWE-PRIL reverse signalling suppresses sympathetic axon growth and tissue innervation

**DOI:** 10.1242/dev.165936

**Published:** 2018-11-19

**Authors:** Laura Howard, Erin Wosnitzka, Darian Okakpu, Matthew A. White, Sean Wyatt, Alun M. Davies

**Affiliations:** School of Biosciences, Cardiff University, Museum Avenue, Cardiff, CF10 3AT, UK

**Keywords:** TNF superfamily, Reverse signalling, Sympathetic axons, Tissue innervation, TNFSFM13, TNFSF13

## Abstract

TWE-PRIL is a naturally occurring fusion protein of components of two TNF superfamily members: the extracellular domain of APRIL; and the intracellular and transmembrane domains of TWEAK with no known function. Here, we show that *April*^−/−^ mice (which lack APRIL and TWE-PRIL) exhibited overgrowth of sympathetic fibres *in vivo*, and sympathetic neurons cultured from these mice had significantly longer axons than neurons cultured from wild-type littermates. Enhanced axon growth from sympathetic neurons cultured from *April*^−/−^ mice was prevented by expressing full-length TWE-PRIL in these neurons but not by treating them with soluble APRIL. Soluble APRIL, however, enhanced axon growth from the sympathetic neurons of wild-type mice. siRNA knockdown of TWE-PRIL but not siRNA knockdown of APRIL alone also enhanced axon growth from wild-type sympathetic neurons. Our work reveals the first and physiologically relevant role for TWE-PRIL and suggests that it mediates reverse signalling.

## Abstract (precis)

**Summary:** *In vivo* and *in vitro* studies of superior cervical ganglion neurons of *April*^−/−^ mice reveal that TWE-PRIL is a physiological regulator of NGF-promoted sympathetic axon growth, acting as a reverse signalling receptor.

## INTRODUCTION

Key to understanding how functional neural circuits are established in development is the identification and characterization of the signals that regulate the growth, branching and disposition of neural processes. A great deal has been learned about the nature of these signals and how they operate by studying neurons of the vertebrate peripheral nervous system (PNS), not least because of the ease with which PNS neurons can be cultured and the ease with which the innervation of various tissues can be studied *in vivo*. Pre-eminent among the signals that regulate the growth of neural processes is the nerve growth factor (NGF) family of neurotrophins (Huang and Reichardt, 2001). These secreted proteins are generally synthesized in the periphery by innervated tissues and have two main functions in PNS neuron development: they promote neuron survival and stimulate the growth and branching of axons within innervated tissues. As such, they play a key role in regulating neuron number and tissue innervation (Davies, 2003; Glebova and Ginty, 2004).

In addition to neurotrophins and other neurotrophic factors, members of the tumour necrosis factor superfamily (TNFSF) have recently been recognized to be physiologically relevant regulators of axon growth during development. The 19 members of this superfamily are best understood for their many crucial functions in the development and function of the immune system, but are increasingly recognized as having diverse roles in other tissues and organs (Hehlgans and Pfeffer, 2005). Several TNFSF members have been shown to modulate axon growth, generally without affecting neuronal survival. For example, in certain populations of neurons, GITRL (TNFSF18) and APRIL (a proliferating-inducing ligand, TNFSF13) enhance axon growth (McWilliams et al., 2017; O'Keeffe et al., 2008; Osório et al., 2014), whereas RANKL (TNFSF11) and LIGHT (TNFSF14) suppress the axon growth-promoting actions of neurotrophins (Gavalda et al., 2009; Gutierrez et al., 2013). Members of the TNFSF are type II transmembrane glycoproteins that are active both as membrane-integrated ligands and as soluble ligands after cleavage from the cell membrane. They exert their effects by binding to members of the tumour necrosis factor receptor superfamily (TNFRSF) (Hehlgans and Pfeffer, 2005). In addition to functioning as ligands, several membrane-integrated TNFSF members can act as reverse signalling receptors for their respective TNFRSF partners, which function as activating ligands (Sun and Fink, 2007). Recently, TNFSF reverse signalling has been shown to be physiologically relevant for the regulation of axon growth and tissue innervation in the developing PNS. For example, TNFR1-activated TNF reverse signalling (Kisiswa et al., 2013, 2017; Wheeler et al., 2014) and CD40-activated CD40L reverse signalling (McWilliams et al., 2015) enhance axon growth and promote the innervation of certain tissues *in vivo*. CD40-activated CD40L reverse signalling also enhances the growth and branching of excitatory neuron dendrites, and suppresses the growth and branching of inhibitory neuron dendrites in the developing CNS (Carriba and Davies, 2017).

APRIL is best characterized for its role in regulating lymphocyte survival and activation (Schneider, 2005). In the developing nervous system, soluble APRIL enhances axon growth from cultured hippocampal pyramidal neurons and midbrain dopaminergic neurons, and *in vivo* studies have revealed that the initial nigrostriatal projection is significantly compromised in *April*^−/−^ embryos (McWilliams et al., 2017; Osório et al., 2014). The *April* gene (*Tnfsf13* – Mouse Genome Informatics) is adjacent to the gene that encodes another member of the TNFSF, TWEAK (TNF-like weak inducer of apoptosis, TNFSF12). A differentially spliced hybrid transcript is translated into a protein, TWE-PRIL, that comprises the extracellular domain of APRIL, and the cytoplasmic and transmembrane domains of TWEAK (Kolfschoten et al., 2003; Pradet-Balade et al., 2002). Although T-cells and monocytes express TWE-PRIL and the protein has been shown to stimulate cycling lymphoma cell lines (Pradet-Balade et al., 2002), no biological function has yet been ascribed to this protein *in vivo*.

The sympathetic neurons of the rodent superior cervical ganglion (SCG) are one of the most extensively studied populations of neurons in the developing PNS. These neurons innervate cranial tissues and become dependent on NGF for survival shortly after their axons reach and ramify within their target tissues (Davies, 2009; Glebova and Ginty, 2005). Our current *in vivo* and *in vitro* studies of SCG neurons of *April*^−/−^ mice (which lack APRIL and TWE-PRIL), together with siRNA transfection in wild-type neurons to selectively knock down expression of particular proteins have revealed the first physiologically relevant role for TWE-PRIL. This functions as a suppressor of NGF-promoted sympathetic axon growth and accomplishes this role via a novel signalling mechanism; TWE-PRIL-mediated reverse signalling activated by one of the canonical APRIL receptors, BCMA (TNFRSF17), functioning as the ligand.

## RESULTS

### Expression of APRIL, TWEAK, TWE-PRIL and BCMA in SCG neurons

To begin to examine whether TWE-PRIL and/or APRIL play a role in developing sympathetic neurons, we used RT-qPCR to assess the expression of *Twe-pril* (*Tnfsfm13* – Mouse Genome Informatics) and *April* mRNAs in SCG dissected from mice at stages throughout embryonic and postnatal development. We also measured the levels of transcripts encoding BCMA (TNFRSF17) and TACI (TNFRSF13B), the TNFRSF members that bind APRIL/TWE-PRIL (Bossen and Schneider, 2006). For completeness, we also measured the levels of *Tweak* mRNA in the developing SCG. To assess expression of *Twe-pril* mRNA, we used primers that bind either side of the junction of the regions encoding the APRIL and TWEAK components of this fusion protein. The relative levels of expression of these transcripts were quantified relative to their levels in adult spleen.

**Fig. 1.**
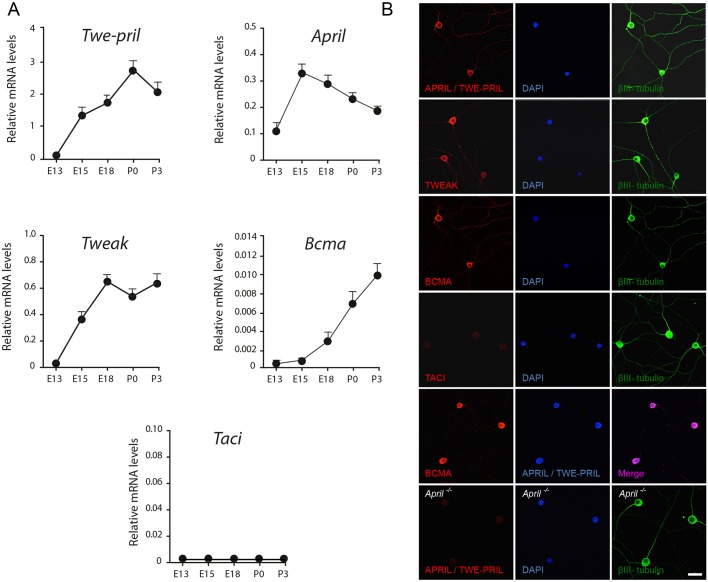
**Developing SCG neurons express APRIL/TWE-PRIL, TWEAK and BCMA.** (A) Graphs of the levels of *Twe-pril*, *April*, *Tweak*, *Bcma* and Taci mRNAs in the SCG of E13, E15, E18, P0 and P3 mice relative to the geometric mean of reference mRNAs for glyceraldehyde phosphate dehydrogenase and succinate dehydrogenase. Data are expressed relative to the levels in adult spleen. (B) Images of dissociated P0 SCG cultures incubated for 16 h in medium containing 0.1 ng/ml NGF and 25 µM Boc-D-FMK, and triple labelled with anti-βIII tubulin antibody, the nuclear marker DAPI and antibodies that bind either APRIL/TWE-PRIL, BCMA, TWEAK or TACI. Cultures of wild-type SCG neurons double-labelled with anti-APRIL/TWE-PRIL and anti-BCMA, and the no anti-APRIL/TWE-PRIL staining controls of cultures established from *April*^−/−^ mice are shown below these panels. Scale bar: 20 µm.

*Twe-pril*, *April*, *Tweak* and *Bcma* mRNAs were reliably and consistently detected in the developing SCG. *April* and *Bcma* mRNAs were first detected at E13, when the SCG becomes anatomically discernible and the earliest axons are growing to their targets. *Twe-pril* and *Tweak* mRNAs were first detected at E15, which is a day after the earliest axons reach their targets and have started to grow and branch within their targets (Fig. 1A). *Taci* mRNA was barely detectable and not accurately quantifiable at any of the embryonic and postnatal ages studied.

To confirm expression of the encoded proteins, we visualized these proteins by immunocytochemistry in dissociated SCG cultures established from P0 mice. TWE-PRIL and APRIL were localized using an antibody that recognizes an epitope in the extracellular domain of both TWE-PRIL and APRIL. This antibody strongly labelled virtually all SCG neurons (Fig. 1B). Labelling was not observed in cultures established from P0 *April*^−/−^ mice (Fig. 1), confirming the specificity of this antibody. Virtually all SCG neurons were also labelled by anti-BCMA and by anti-TWEAK. Labelling with anti-TACI antibodies was barely above background. In double labelling experiments, the neurons were co-labelled by anti-APRIL/TWE-PRIL and anti-BCMA. These studies suggest that SCG neurons co-express TWE-PRIL/APRIL and BCMA.

### Increased *in vivo* growth of sympathetic fibres in *April*^−/−^ mice

Given the evidence that APRIL promotes the early growth of axons from at least two kinds of CNS neurons (McWilliams et al., 2017; Osório et al., 2014) and our current demonstration that developing SCG neurons express TWE-PRIL/APRIL, we investigated whether absence of TWE-PRIL/APRIL affects the projection and early ramification of sympathetic axons in their targets by comparing the sympathetic innervation of *April*^−/−^ embryos (which lack APRIL and TWE-PRIL) and *April*^+/+^ littermates. Immunolabelling-enabled three-dimensional imaging of solvent-cleared organs (iDISCO) (Renier et al., 2014) is particularly appropriate for examining this, as it facilitates imaging the sympathetic innervation of SCG targets in cleared heads at a stage of development when sympathetic fibres are growing and branching within their targets. Sympathetic fibres were labelled with an antibody against tyrosine hydroxylase (TH), a rate-limiting enzyme of noradrenaline synthesis. These studies were carried out at E16, which is 2 days after the earliest sympathetic fibres have reached their targets and have started to ramify in these targets, but is before the sympathetic innervation becomes too dense to reliably quantify with this technique.

**Fig. 2.**
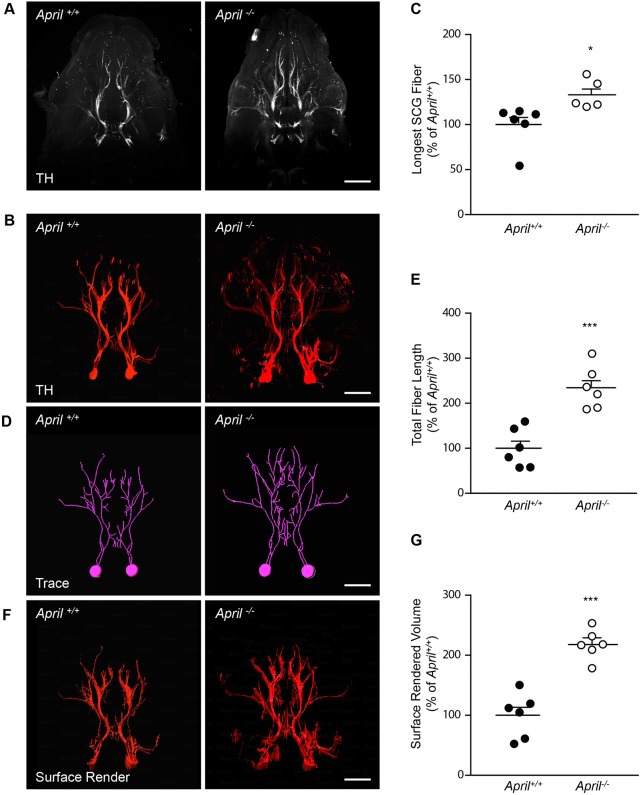
**Increased growth of SCG fibres in E16 *April*^−/−^ embryos.** (A) Representative collapsed *z*-stack light sheet fluorescent microscope images of *April*^+/+^ and *April*^−/−^ heads of iDISCO preparations stained for TH. (B) Representative Bitplane Imaris reconstructions of light sheet *z*-stacks of *April*^+/+^ and *April*^−/−^ heads viewed in the transverse plane. (C) Scatter bar chart of the lengths of the longest SCG fibres measured in Bitplane Imaris reconstructions (mean and individual data points, *n*=6 separate embryos of each genotype, *P<0.05, *t*-test). (D) Representative manual tracings of rostral SCG fibres made from Bitplane Imaris reconstructions of *April*^+/+^ and *April*^−/−^ heads viewed in the transverse plane. (E) Scatter bar chart of the combined lengths of rostral SCG fibres measured in Bitplane Imaris reconstructions (mean and individual data points, *n*=6 separate embryos of each genotype, ***P<0.001, *t*-test). (F) Representative images of SCG fibre reconstruction using an automatic surface rendering programme based on Bitplane Imaris reconstructions of *April*^+/+^ and *April*^−/−^ heads viewed in the transverse plane. (G) Scatter bar chart representing the total surface volume of rostral SCG fibres (proportional to total fibre length) in reconstructions generated by the Imaris Surface Creation module (mean and individual data points, *n*=6 separate embryos of each genotype, ***P<0.001, *t*-test). Scale bars: 1 mm.

The iDISCO preparations were visualized by light sheet fluorescent microscopy at optical sections of 5 µm (between 600 and 800 optical sections per animal, which included all rostrally directed fibres from both SCG). Individual optical sections were assembled into three-dimensional reconstructions of sympathetic fibres emanating from the SCG using Bitplane Imaris software (Bitplane Scientific Solutions, Zurich, Switzerland), which allows topographic manipulation of the reconstructions for accurate fibre tracing and measurement. Examination of collapsed *z*-stack light sheet images (Fig. 2A) and Bitplane Imaris reconstructions (Fig. 2B) showed that the gross anatomical disposition of individual fibres in whole heads of *April*^+/+^ and *April*^−/−^ mice were similar at E16. However, measurements of the lengths of the longest SCG fibres (which terminate in nasal tissue at this stage) in Bitplane Imaris reconstructions revealed significantly longer fibres in *April*^−/−^ mice compared with *April*^+/+^ littermates (Fig. 2C). Bitplane Imaris reconstructions facilitated semi-manual tracing of individual SCG fibres (Fig. 2D). The small number of caudally directed and short local SCG fibres were excluded from these tracings because the full extent of caudally directed fibres could not be followed because several of these had been severed when heads were collected for iDISCO preparations and because of the complexity of local SCG fibres. Measurements taken from these manual tracings revealed a highly significant, twofold increase in the combined lengths of fibres in E16 *April*^−/−^ mice compared with *April*^+/+^ littermates (Fig. 2E). All analysis was carried out blind. Importantly, when quantification was carried out using a surface rendering programme that required no observer input following manual threshold optimization (Fig. 2F), *April*^−/−^ heads still exhibited a highly significant increase in total sympathetic innervation compared with *April*^+/+^ littermates (Fig. 2G).

To exclude the possibility that these results may reflect differences in the level of expression of TH between the SCG neurons of *April*^+/+^ and *April*^−/−^ mice, we used western blotting to estimate the relative levels of TH in the SCG of these mice. There was no significant difference in TH levels between the SCG of *April*^+/+^ and *April*^−/−^ mice at E16 (Fig. S1). In summary, our studies suggest that SCG sympathetic fibres in *April*^−/−^ embryos grow longer than in *April*^+/+^ littermates.

### Increased *in vitro* growth of axons from *April*^−/−^ SCG neurons

**Fig. 3.**
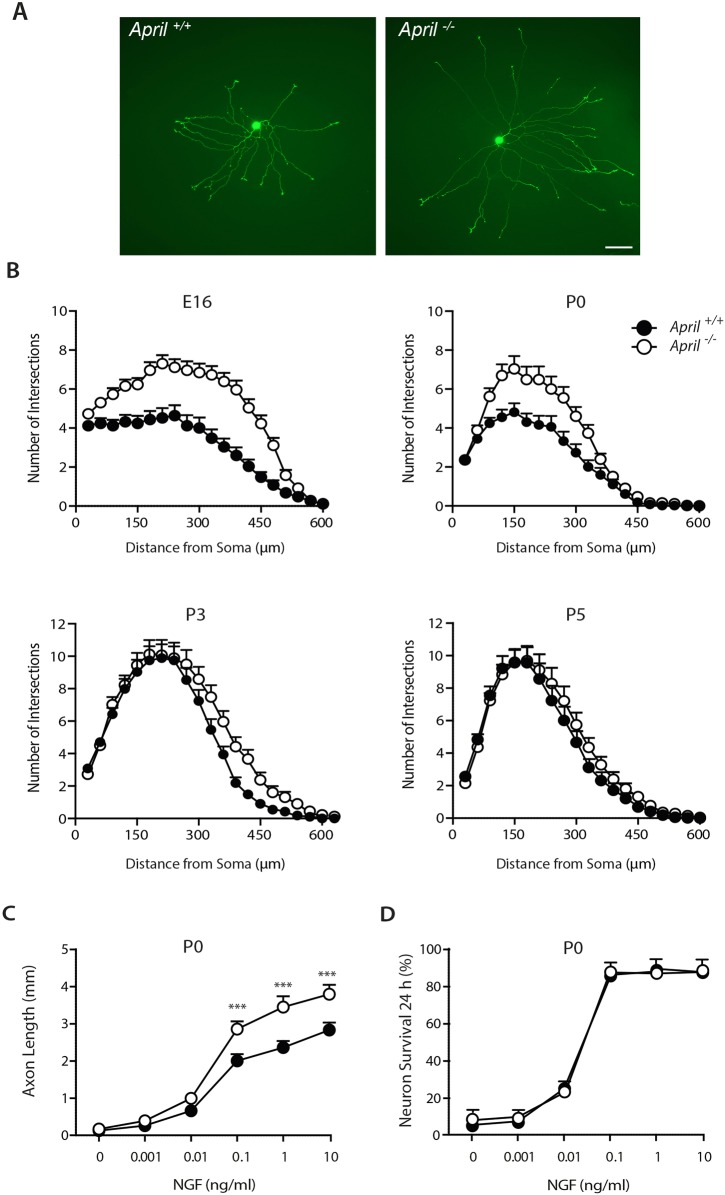
**Enhanced NGF-promoted axon growth from cultured *April*^−/−^ SCG neurons.** (A) Micrographs of representative calcein-labelled P0 *April*^+/+^ and *April*^−/−^ SCG neurons incubated for 16 h in medium containing 0.1 ng/ml NGF and 25 µM Boc-D-FMK. Scale bar: 100 µm. (B) Sholl plots of E16, P0, P3 and P5 SCG neurons incubated for 16 h in medium containing 0.1 ng/ml NGF and 25 µM Boc-D-FMK. Mean±s.e.m. of data from over 50 neurons per condition. (C) Graphs of total axon length of the arbors of P0 *April*^+/+^ and *April*^−/−^ SCG neurons incubated for 16 h in medium containing 25 µM Boc-D-FMK and different concentrations of NGF. Mean±s.e.m. of data from more than 50 neurons per condition (***P<0.001, statistical comparison between *April*^+/+^ and *April*^−/−^, ANOVA). (D) Graph of the survival of P0 *April*^+/+^ and *April*^−/−^ SCG neurons incubated for 24 h in medium containing different concentrations of NGF. Mean±s.e.m. of data from three separate experiments.

To ascertain whether the long sympathetic fibre phenotype observed in *April*^−/−^ embryos *in vivo* is replicated by sympathetic neurons cultured from these embryos, we compared the size of the axon arbors of SCG neurons cultured from *April*^+/+^ and *April*^−/−^ mice. We established very low density dissociated cultures from E16, P0, P3 and P5 *April*^+/+^ and *April*^−/−^ littermates in culture medium supplemented with 0.1 ng/ml NGF to stimulate axon growth and 25 μM of pancaspase inhibitor Boc**^_^**D**^_^**FMK to prevent apoptosis. After 16 h incubation, the neurons were labelled with the fluorescent vital dye calcein-AM, and images of individual neurons were acquired. In these short-term cultures, all neurites are structurally axons (Lein et al., 1995). Neurons cultured from *April*^−/−^ mice had larger axon arbors than those grown from *April*^+/+^ mice, as shown by representative neurons in P0 cultures (Fig. 3A). These differences and how they change in development are clearly illustrated in Sholl plots, which provide a graphic illustration of axon length and branching with distance from the cell body (Sholl, 1953). The differences in axon arbor size between *April*^−/−^ and *April*^+/+^ neurons were greatest in cultures established at E16 and P0. Differences were negligible in P3 cultures and were not discernable in P5 cultures (Fig. 3B).

NGF dose responses carried out at E16, P0 and P3 in the presence of the pancaspase inhibitor Boc**^_^**D**^_^**FMK revealed that the difference in total axon length in the neuron arbors between *April*^−/−^ and *April*^+/+^ neurons increased with NGF concentration. As shown in P0 cultures, there were no differences in total axon length in the absence of NGF, and the differences between *April*^−/−^ and *April*^+/+^ neurons increased with increasing NGF concentration up to 10 ng/ml and (the highest concentration of NGF used) (Fig. 3C). Very similar differences were observed in E16 cultures and a similar trend was observed in P3 cultures, although the differences were not as great (not shown).

Although there were significant differences in NGF-promoted axon growth between the SCG neurons of *April*^+/+^ and *April*^−/−^ mice, there were no significant differences in NGF-promoted survival. In the absence of Boc**^_^**D**^_^**FMK, overlapping NGF survival dose responses were obtained from SCG neurons of *April*^+/+^ and *April*^−/−^ mice, as illustrated for P0 cultures (Fig. 3D). Overlapping NGF survival dose responses were also observed in cultures of SCG neurons of *April*^+/+^ and *April*^−/−^ littermates set up at E16 and P3 (not shown).

In summary, these findings show that NGF-promoted axon growth, but not survival, is significantly greater from *April*^−/−^ SCG neurons, compared with *April*^+/+^ SCG neurons. The enhanced response to NGF occurs over a window of perinatal development when axons are ramifying in their targets under the influence of target-derived NGF. Because SCG neurons cultured from *April*^−/−^ mice mirror the *in vivo* phenotype, we were able to undertake additional *in vitro* studies to clarify the mechanism underlying this phenotype.

### siRNA knockdown of TWE-PRIL/APRIL, but not APRIL alone, increases sympathetic axon growth

**Fig. 4.**
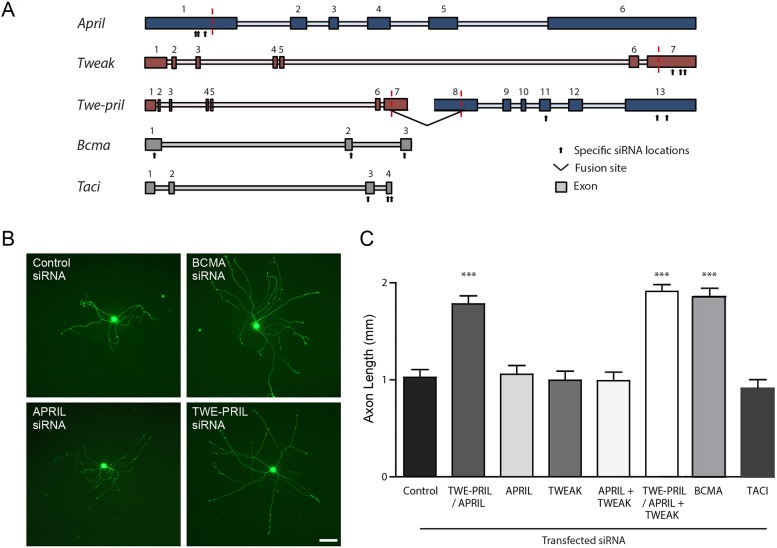
**siRNA knockdown of either TWE-PRIL or BCMA, but not APRIL alone, enhances NGF-promoted axon growth from SCG neurons.** (A) Schematic diagram of the intron/exon structure of the *April*, *Tweak*, *Bcma* and *Taci* genes, and the regions of the *April* (blue) and *Tweak* (red) genes from which the primary *Twe-pril* transcript is generated. The fusion sites of *April* and *Tweak* that generate the primary *Twe-pril* transcript are indicated by vertical red lines. Exons are numbered and indicated by boxes. The sites to which sets of siRNA bind are indicated by arrows. (B) Micrographs of representative P0 SCG neurons that were co-transfected with a GFP expression plasmid and either control scrambled siRNA, BCMA siRNA, APRIL-specific siRNA or a siRNA that knocks down TWE-PRIL and APRIL. The neurons were incubated for 16 h after transfection in medium containing 1 ng/ml NGF and 25 µM Boc-D-FMK. Scale bar: 100 µm. (C) Bar chart of total axon length of the arbors of P0 SCG neurons transfected with a GFP expression plasmid plus: control scrambled siRNA, siRNA that knocks down TWE-PRIL plus APRIL, siRNA that selectively knocks down either APRIL, TWEAK, BCMA or TACI without targeting TWE-PRIL expression, siRNA that knocks down APRIL and TWEAK, and siRNA that knocks down TWE-PRIL plus APRIL and TWEAK. Mean±s.e.m. of data from over 50 neurons per condition (***P<0.001, comparison with control siRNA, ANOVA).

To complement and extend our *in vitro* studies of NGF-promoted axon growth from SCG neurons cultured from *April*^+/+^ and *April*^−/−^ mice, we used siRNA to knock down expression of TWE-PRIL, APRIL, TWEAK, BCMA and TACI in SCG neurons cultured from wild-type mice. We co-transfected P0 SCG neurons with specific siRNA oligonucleotides, together with a GFP expression vector to identify and label the transfected neurons. The neurons were transfected by electroporation, after which they were plated in NGF-containing medium and were imaged 16 h later. We used combinations of three different specific 21-mer siRNA oligonucleotides that have been individually validated by the manufacturer to knock down expression of the target protein in cell lines. Although it was not possible to knock down TWE-PRIL without knocking down either APRIL or TWEAK, by using siRNAs that target the parts of *April* and *Tweak* mRNAs that do not encode part of TWE-PRIL it was possible to knock down either APRIL or TWEAK without affecting TWE-PRIL expression (Fig. 4A). To control for non-specific effects of siRNA transfection, we transfected SCG neurons with a 21-mer double-stranded RNA oligonucleotide that has a scrambled sequence.

We used immunocytochemistry to confirm selective knockdown of these proteins in transfected neurons. BCMA siRNAs greatly reduced BCMA immunoreactivity, TWEAK siRNAs eliminated TWEAK immunoreactivity, and either APRIL siRNAs or APRIL/TWE-PRIL siRNAs eliminated APRIL/TWE-PRIL immunoreactivity (Fig. S2). TWEAK siRNAs did not affect APRIL/TWE-PRIL immunoreactivity and APRIL siRNAs did not affect TWEAK immunoreactivity (Fig. S3).

Neurons transfected with siRNAs that reduce expression of TWE-PRIL and APRIL (siRNAs that targeted sequences in exons 4 and 6 of the *April* gene, equivalent to exons 11 and 13 of the *Twe-pril* primary transcript, Fig. 4A) had significantly longer axons than those of neurons transfected with control RNA oligonucleotides (Fig. 4B,C). In contrast, neurons transfected with siRNAs that selectively knock down APRIL expression without affecting TWE-PRIL expression (siRNAs that targeted sequences in exon 1 of the *April* gene, Fig. 4A) had axons that were no longer than those of neurons transfected with control RNA oligonucleotides (Fig. 4B,C). Likewise, siRNA that specifically knocked down expression of TWEAK without affecting TWE-PRIL expression (siRNAs that targeted sequences in exon 7 of the *Tweak* gene) had no significant effect on axon growth (Fig. 4C). Knocking down expression of both APRIL and TWEAK without affecting TWE-PRIL expression (co-transfecting neurons with the siRNAs that selectively target APRIL plus siRNAs that target TWEAK) also had no significant effect on axon growth. However, co-transfecting neurons with the siRNAs that reduce expression of TWE-PRIL and APRIL plus the siRNAs that target TWEAK had had significantly longer axons than those of neurons transfected with control RNA oligonucleotides (Fig. 4C). Taken together, these findings suggest that TWE-PRIL, but neither APRIL nor TWEAK, participates in the suppression of NGF-promoted axon growth from cultured SCG neurons. This in turn suggests that absence of TWE-PRIL, rather than absence of APRIL, underlies the *in vivo* sympathetic phenotype of *April*^−/−^ embryos.

In accordance with differences in the expression of BCMA and TACI (Fig. 1A), siRNAs that specifically targeted BCMA, but not siRNAs that targeted TACI, significantly increased axon growth from transfected SCG neurons (Fig. 4B,C). This suggests that BCMA, but not TACI, participates in the suppression of NGF-promoted axon growth from developing SCG neurons.

### Rescue of the *in vitro* phenotype of *April*^−/−^ SCG neurons by expressing TWE-PRIL

The above findings suggest that the interaction between TWE-PRIL and BCMA suppresses NGF-promoted axon growth. However, these findings do not indicate the direction of signalling: either TWE-PRIL-activated BCMA-mediated forward signalling or BCMA-activated TWE-PRIL-mediated reverse signalling. To distinguish between these alternatives, we asked whether or not soluble APRIL was sufficient to reverse the enhanced NGF-promoted axon growth from *April*^−/−^ SCG neurons (which would be consistent with forward signalling) or whether expression of membrane integrated TWE-PRIL is required.

**Fig. 5.**
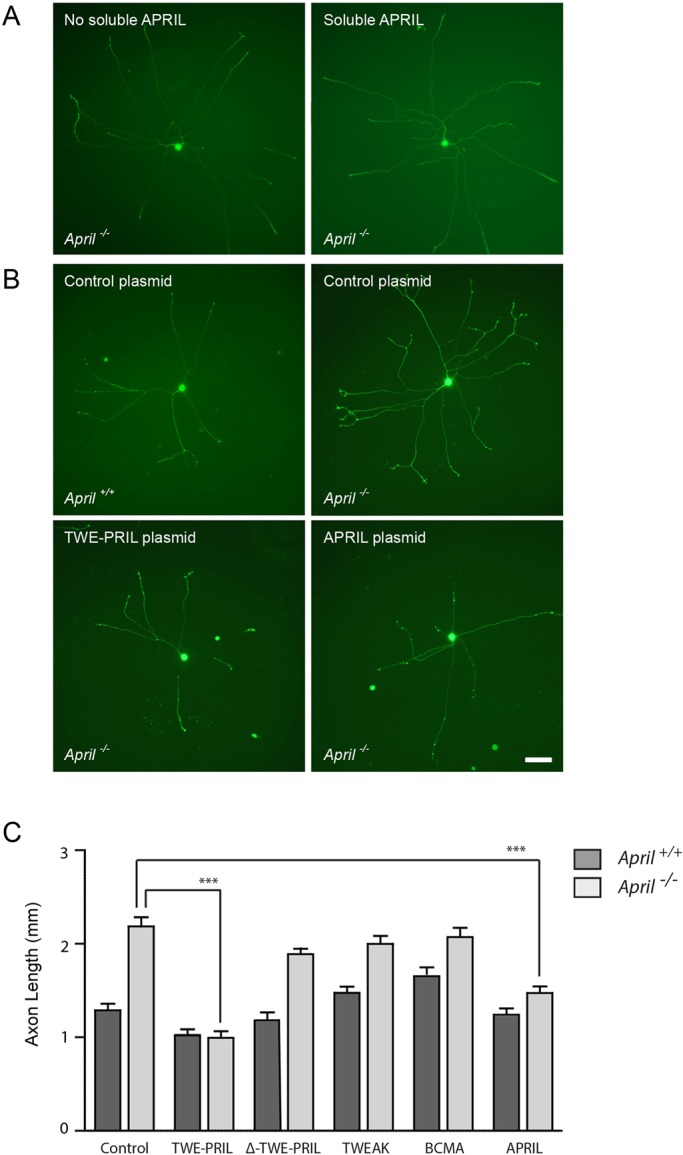
**Rescue of *in vitro* phenotype of *April*^−/−^ SCG neurons transfected with either full-length TWE-PRIL or full-length APRIL expression plasmids but not by transfection of truncated TWE-PRIL, TWEAK or BCMA expression plasmids or treatment with soluble APRIL.** (A) Images of representative calcein-labelled P0 *April*^−/−^ SCG neurons incubated for 16 h in medium containing 0.1 ng/ml NGF and 25 µM Boc-D-FMK with and without 100 ng/ml soluble recombinant APRIL. (B) Images of representative P0 SCG neurons of *April*^+/+^ and *April*^−/−^ mice that were co-transfected with a GFP expression plasmid plus either an empty control plasmid or plasmids that express either full-length (FL) TWE-PRIL or FL-APRIL. The neurons were incubated for 16 h after transfection in medium containing 1 ng/ml NGF and 25 µM Boc-D-FMK. Scale bar: 100 µm. (C) Bar chart of total axon length of SCG neurons from *April*^+/+^ and *April*^−/−^ mice that were co-transfected with a GFP expression plasmid plus either an empty control plasmid or plasmids that express either full-length APRIL, full-length TWE-PRIL, truncated TWE-PRIL that lacks a cytoplasmic domain (ΔTWE-PRIL), BCMA and TWEAK. Mean±s.e.m. of data from over 50 neurons per condition (***P<0.001, ANOVA).

The axon arbors of NGF-supplemented P0 *April*^−/−^ SCG neurons were very similar with or without soluble recombinant APRIL in the culture medium (Fig. 5A). This suggests that soluble APRIL is unable to rescue the enhanced NGF-promoted axon growth phenotype of cultured *April*^−/−^ SCG neurons. In contrast, the axon arbors of NGF-supplemented P0 *April*^−/−^ SCG neurons transfected with a full-length TWE-PRIL expression plasmid were significantly shorter than those of control-transfected *April*^−/−^ SCG neurons (neurons transfected with an empty expression plasmid) and were not significantly different from control-transfected *April*^+/+^ SCG neurons (Fig. 5B,C). This suggests that expression of full-length TWE-PRIL completely rescued the enhanced NGF-promoted axon growth phenotype of *April*^−/−^ SCG neurons. These findings are consistent with the idea that TWE-PRIL-mediated reverse signalling suppresses NGF-promoted axon growth.

A wealth of evidence suggests that the intracellular domain of TNFSF members that are able to mediate reverse signalling is essential for this mode of signalling (Sun and Fink, 2007). To test the importance of the intracellular domain of TWE-PRIL for reverse signalling, we transfected neurons with a plasmid that expresses a truncated form of TWE-PRIL that lacks the entire intracellular domain derived from TWEAK apart from the initiation codon of this type II glycoprotein but retains the transmembrane domain derived from TWEAK and the extracellular domain derived from APRIL (ΔTWE-PRIL). The size of the axon arbors of NGF-supplemented P0 *April*^−/−^ SCG neurons transfected with this expression plasmid were not significantly different from those of control-transfected *April*^−/−^ SCG neurons (Fig. 5C). Expression of neither full-length TWEAK nor full-length BCMA were able to substitute for full-length TWE-PRIL in suppressing enhanced NGF-promoted axon growth from *April*^−/−^ SCG neurons (Fig. 5C).

Interestingly, transfecting *April*^−/−^ SCG neurons with a plasmid that expresses full-length APRIL was able to prevent enhanced NGF-promoted axon growth from *April*^−/−^ SCG neurons. This suggests that overexpressed APRIL is able to mediate reverse signalling and is able to substitute for TWE-PRIL in suppressing NGF-promoted axon growth from *April*^−/−^ SCG neurons.

### Soluble APRIL replicates the *in vitro* phenotype of *April*^−/−^ SCG neurons

**Fig. 6.**
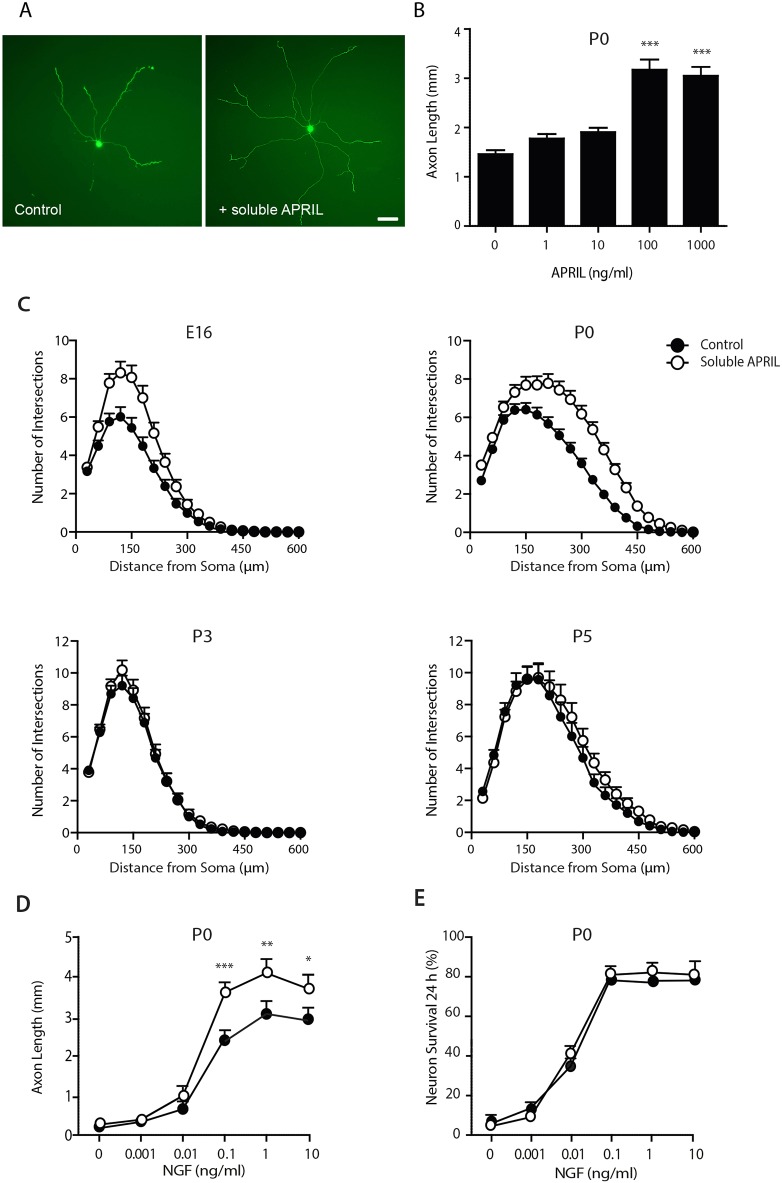
**Soluble APRIL enhances NGF-promoted axon growth from SCG neurons.** (A) Images of representative calcein-labelled P0 *April*^+/+^ SCG neurons incubated for 16 h in medium containing 0.1 ng/ml NGF and 25 µM Boc-D-FMK with and without 100 ng/ml soluble recombinant APRIL. Scale bar: 100 µm. (B) Bar charts of total length of axons in the arbors of P0 SCG neurons grown for 16 h in medium containing 0.1 ng/ml NGF, 25 µM Boc-D-FMK and different concentrations of APRIL. Mean±s.e.m. of data from over 50 neurons per condition (***P<0.001, statistical comparison with no APRIL). (C) Sholl plots of E16, P0, P3 and P5 *April*^+/+^ SCG neurons incubated for 16 h in medium containing 0.1 ng/ml NGF and 25 µM Boc-D-FMK, with and without 100 ng/ml APRIL. (D) Graphs of total axon length in the arbors of P0 *April*^+/+^ SCG neurons incubated for 16 h in medium containing Boc-D-FMK and different concentrations of NGF, with and without 100 ng/ml APRIL. Mean±s.e.m. of data from over 50 neurons per condition (*P<0.05, **P<0.01, ***P<0.001, statistical comparison of neurons grown with and without APRIL, ANOVA). (E) Graph of the survival of P0 SCG neurons incubated for 24 h in medium containing different concentrations of NGF with and without 100 ng/ml APRIL. Mean±s.e.m. of data from three separate experiments.

Although the above findings are consistent with the operation of BCMA-activated TWE-PRIL-mediated reverse signalling, in order to provide an additional test that signalling occurs in this direction, we studied the effects of soluble recombinant APRIL on NGF-promoted axon growth from wild-type SCG neurons. We reasoned that soluble APRIL should compete with endogenous membrane integrated TWE-PRIL for binding to BCMA. This should interfere with endogenous BCMA-activated TWE-PRIL-mediated reverse signalling, but should not inhibit BCMA-mediated forward signalling. Soluble APRIL caused a dose-dependent increase in NGF-promoted axon length, reaching a plateau at 100 ng/ml APRIL (Fig. 6A,B). The period of development over which soluble APRIL treatment enhanced NGF-promoted axon growth from wild-type SCG neurons was similar to the period of development over which SCG neurons cultured from *April*^−/−^ mice displayed increased responsiveness to NGF. Sholl plots of NGF-supplemented E16, P0, P3 and P5 wild-type SCG neurons grown with and without soluble APRIL showed that the enhancement of NGF-promoted axon growth by APRIL was greatest in E16 and P0 cultures (Fig. 6C). Moreover, the magnitude by which APRIL enhanced NGF-promoted axon growth from wild-type SCG neurons was very similar to the magnitude of NGF-promoted axon growth from SCG neurons cultured from *April*^−/−^ mice, compared with the magnitude of NGF-promoted axon growth from SCG neurons cultured from *April*^+/+^ mice (compare Figs 3B and 6C). The influence of soluble APRIL on NGF-promoted axon growth from wild-type SCG neurons was also related to NGF concentration. Although APRIL had no effect on axon growth from SCG neurons grown with low levels of NGF, it progressively enhanced NGF-promoted axon growth with increasing NGF concentration (Fig. 6D), as is the case with SCG neurons of *April*^−/−^ mice compared with SCG neurons of *April*^+/+^ mice. As is the case with absence of APRIL, treatment with soluble APRIL had no effect on NGF-promoted survival. In the absence of Boc**^_^**D**^_^**FMK, overlapping NGF survival dose responses were observed from P0 SCG neurons grown with and without APRIL (Fig. 6E). Taken together, the above results indicate that soluble APRIL enhances NGF-promoted axon growth from wild-type SCG neurons, but not NGF-promoted survival, over the same period of development and to the same extent as a null mutation in the *April* gene.

### TWE-PRIL reverse signalling suppresses axonal growth by supressing ERK1/ERK2 activation in response to NGF

**Fig. 7.**
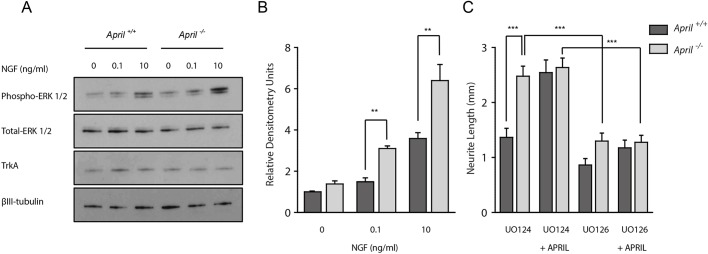
**NGF-stimulated ERK activation is enhanced in *April*^−/−^ neurons and inhibition of ERK activation eliminates the differences in axon growth between *April*^−/−^ and *April*^+/+^ neurons.** (A) Representative western blots probed for phospho-ERK1/ERK2, total ERK1/ERK2, TrkA and β-III tubulin of lysates of P0 SCG neurons of *April*^−/−^ and *April*^+/+^ mice that had been incubated with 25 µM Boc-D-FMK for 12 h before either 30 min treatment with NGF (0.1 or 10 ng/ml) or no treatment. (B) Densitometry of three separate experiments (mean±s.e.m. of phospho-ERK1/ERK2 relative to total ERK1/ERK2). **P<0.01, statistical comparison indicated. (C) Bar charts of total length of axons in the arbors of P0 SCG neurons of *April*^−/−^ and *April*^+/+^ mice that were plated in medium containing either 10 µM U0126 or 10 µM U0124 and were treated with either 0.1 ng/ml NGF or 0.1 ng/ml NGF plus 100 ng/ml APRIL 1 h after plating. Axon arbors were imaged 16 h later. Mean±s.e.m. of data from over 50 neurons per condition (***P<0.001, statistical comparison indicated, ANOVA).

Because activation of the MEK/ERK pathway contributes to the axon growth response to NGF (Gómez and Cohen, 1991; Goold and Gordon-Weeks, 2005; O'Keeffe et al., 2008) and is an essential step in the axon growth response of sympathetic neurons to TNFR1-activated TNFα-mediated reverse signalling (Kisiswa et al., 2017), we investigated whether TWE-PRIL/APRIL modulates ERK activation by NGF and whether this plays a role in axon growth suppression by TWE-PRIL reverse signalling. We began by investigating whether the expression of TWE-PRIL/APRIL influences ERK activation by NGF. For these studies, we used western blotting to compare ERK activation in response to NGF in SCG neurons cultured at high density from *April*^+/+^ and *April*^−/−^ mice. These studies revealed that the level of phospho-ERK1/ERK2 relative to total ERK1/ERK2 was significantly greater in NGF stimulated P0 SCG neurons cultured from *April*^−/−^ mice compared with those cultured from *April*^+/+^ littermates (Fig. 7A,B).

To investigate whether suppression of NGF-stimulated ERK1/ERK2 activation by TWE-PRIL reverse signalling contributes to axon growth suppression, we studied the effect of U0126, a selective MEK1/MEK2 inhibitor that interferes with MEK1/MEK2 dependent activation of ERK1/ERK2, on axon growth from SCG in which TWE-PRIL reverse signalling was either permitted or prevented. In these experiments, P0 SCG neurons from *April*^−/−^ and *April*^+/+^ mice were plated at low density in medium containing either U0126 or U0124, and were treated with NGF after 1 h. Half of the cultures were additionally treated with soluble APRIL at this time to inhibit TWE-PRIL reverse signalling. Neurons in all cultures were imaged 16 h after plating. The control compound U0124 did not affect the marked difference in axon length between neurons cultured from *April*^−/−^ and *April*^+/+^ mice. However, axon length in cultures established from *April*^−/−^ mice was significantly reduced by U0126 to the level observed from *April*^+/+^ neurons cultured with U0124 (Fig. 7C). U0126 also significantly reduced the enhanced axon growth of wild-type neurons treated with APRIL to the level observed in wild-type neurons that had not been treated with APRIL (Fig. 7C). Taken together, these findings suggest that suppression of NGF-stimulated ERK activation contributes to the suppression of axon growth by TWE-PRIL reverse signalling.

We also explored the possibility that differences in the expression of NGF receptors might account for the differences in the activation of ERK1/ERK2 in *April*^−/−^ and *April*^+/+^ SCG neurons by NGF. Western blotting revealed there were no differences in the intensity of the bands corresponding to TrkA (the NGF receptor tyrosine kinase) in SCG neurons from *April*^−/−^ and *April*^+/+^ mice grown with different levels of NGF (Fig. 7A). In addition, qPCR revealed that there were no significant differences in the levels of TrkA mRNA and p75 mRNA (which encodes the common neurotrophin receptor) between *April*^−/−^ and *April*^+/+^ SCG (Fig. S4).

## DISCUSSION

We have shown that *April*-null mice, which lack both APRIL and TWE-PRIL, have significantly longer sympathetic fibres *in vivo* at the stage when sympathetic axons are starting to ramify in their targets under the influence of target-derived NGF. This implies that APRIL and/or TWE-PRIL are physiologically relevant suppressors of sympathetic fibre elongation *in vivo.* The axon arbors of sympathetic neurons cultured from *April*^−/−^ mice were significantly larger than those of wild-type littermates grown in the presence of NGF, but were not significantly different in the absence of NGF. This suggests that APRIL and/or TWE-PRIL suppress NGF-promoted axon growth from developing sympathetic neurons. The difference in NGF-promoted axon growth between SCG neurons of *April*^+/+^ and *April*^−/−^ mice was observed over a window of development from late foetal to early postnatal stages. Moreover, whereas the axon growth response of SCG neurons to NGF was affected by the *April*-null mutation, the survival response of these neurons to NGF was completely unaffected. This suggests that APRIL and/or TWE-PRIL suppress NGF-promoted axon growth, but not NGF-promoted neuron survival. These features echo emerging themes in the influence of the TNFSF on axon growth in the developing PNS. Whereas several TNFSF and TNFRSF members promote or inhibit axon growth, they have no or negligible effect on neuron survival (Gavalda et al., 2009; Gutierrez et al., 2013; Kisiswa et al., 2013; McWilliams et al., 2015; O'Keeffe et al., 2008). The effect of these factors on axon growth is transient. Typically, the neurons begin responding to these factors several days after they have started to respond to neurotrophins, and the developmental window over which they exert their effects is short compared with the extended period over which neurotrophins act. The transient influence of the TNFSF/TNFRSF member on axon growth in the developing PNS generally coincides with the perinatal period when axons are ramifying extensively in their targets. It is therefore likely that the TNFSF plays an important role refining innervation during this crucial period of development.

The enhanced NGF-promoted axon growth phenotype of SCG neurons cultured from *April*^−/−^ mice was replicated by transfecting wild-type SCG neurons with siRNA that knock down expression of both TWE-PRIL and APRIL, but was not observed in SCG neurons transfected with siRNA that selectively knock down either APRIL or TWEAK. This suggests that TWE-PRIL, but neither APRIL nor TWEAK, is responsible for suppressing NGF-promoted axon growth. This in turn suggests that the increased growth of SCG nerve fibre branches in *April*^−/−^ embryos *in vivo* is due to the absence of TWE-PRIL rather than the absence of APRIL.

TWE-PRIL comprises the extracellular ligand-binding domain of APRIL fused to the cytoplasmic and transmembrane domains of TWEAK (Kolfschoten et al., 2003; Pradet-Balade et al., 2002), and binds the same two members of the TNFRSF as APRIL, BCMA and TACI. Several findings suggest that BCMA, rather than TACI, is required for the suppression of NGF-promoted axon growth by TWE-PRIL. First, whereas *Bcma* mRNA is present in developing SCG from E13 onwards, *Taci* mRNA was barely detectable at any age. Second, BCMA immunoreactivity was clearly present in cultured neonatal SCG neurons, whereas TACI immunoreactivity was barely above background. Third, transfecting SCG with siRNA that knock down BCMA enhanced NGF-promoted axon growth as effectively as siRNA knockdown of TWE-PRIL, whereas siRNA directed against TACI had no effect on NGF-promoted axon growth.

Two observations suggest that BCMA and TWE-PRIL modulate axon growth by an autocrine mechanism. First, almost all neonatal SCG neurons were labelled by antibodies to BCMA and TWE-PRIL/APRIL, suggesting that these neurons co-express BCMA and TWE-PRIL/APRIL. Second, all *in vitro* experiments were carried out on SCG neurons cultured at exceptionally low density. As such, the neurons were non-contiguous and were cultured in a relatively large volume of medium. Co-labelling and very low-density culture studies of sympathetic and sensory neurons have likewise concluded that several members of the TNFSF and TNFRSF families are likely to exert their effects on axon growth by an autocrine mechanism. These include enhanced NGF-promoted sympathetic axon growth by GITRL/GITR (O'Keeffe et al., 2008) and CD40L/CD40 (McWilliams et al., 2015), and suppressed BDNF-promoted sensory axon growth by LIGHT/HVEM (Gavalda et al., 2009). In addition to autocrine signalling, the TNFSF/TNFRSF can also regulate axon growth and tissue innervation by a paracrine mechanism, e.g. target-derived TNFR1 enhances sympathetic axon growth by activating TNF reverse signalling in axons (Kisiswa et al., 2013, 2017).

It is likely that autocrine interaction of TWE-PRIL and BCMA suppresses NGF-promoted axon growth by a BCMA-activated TWE-PRIL-mediated reverse signalling mechanism. First, the enhanced NGF-promoted axon growth phenotype of SCG neurons cultured from *April*^−/−^ mice is completely rescued by transfecting the neurons with a TWE-PRIL expression plasmid but not by treating the neurons with soluble APRIL. Because the expressed TWE-PRIL is able to integrate into the membrane it has the potential to mediate reverse signalling. Soluble APRIL, on the other hand, is not membrane integrated and only has the potential to activate BCMA-mediated forward signalling. Second, soluble APRIL enhances NGF-promoted axon growth of wild-type SCG neurons as effectively as deletion of *April/Twe-pril* or siRNA knockdown of TWE-PRIL. The most parsimonious explanation for this result is that soluble APRIL competes with endogenous membrane-integrated TWE-PRIL for binding to endogenous BCMA, and thereby inhibits endogenous BCMA from activating TWE-PRIL-mediated reverse signalling. Furthermore, these experiments also control for the efficacy of the soluble APRIL used in the phenotype rescue experiments.

We showed that enhanced NGF-promoted axon growth from *April*^−/−^ SCG neurons can also be eliminated by transfecting the neurons with an APRIL expression plasmid. This suggests that membrane-integrated APRIL is also able to mediate reverse signalling and can substitute for TWE-PRIL in rescuing the phenotype of *April*^−/−^ SCG neurons *in vitro*. However, siRNA that specifically knocks down APRIL in wild-type SCG neurons and leaves TWE-PRIL unaffected does not affect NGF-promoted axon growth. This suggests that endogenous APRIL reverse signalling does not normally contribute to the regulation of NGF-promoted axon growth. This apparent discrepancy may be because endogenous APRIL may be expressed at a much lower level than TWE-PRIL in developing SCG neurons. Moreover, it has been shown in HEK293 cells that APRIL is cleaved in the Golgi apparatus by a furin convertase to generate a biologically active secreted protein (Lopez-Fraga et al., 2001). This implies that APRIL is not expressed at the cell surface and therefore could not mediate reverse signalling. Because APRIL expression in our transfection experiments is under the control of a strong promoter, APRIL may have been expressed at levels that exceed the capacity of the Golgi apparatus to process it. Although our data do not support a role of endogenous APRIL-mediated reverse signalling in modulating NGF-promoted axon growth in SCG neurons, we cannot exclude the possibility that APRIL reverse signalling contributes to the regulation of sympathetic axon growth *in vivo*.

Contrary to the view that APRIL is produced solely as a secreted ligand (Lopez-Fraga et al., 2001), more recent studies have reported APRIL expression at the cell membrane of monocyte and macrophage cell lines, and primary macrophages (Lee et al., 2010a,b). Furthermore, it has been reported that TACI-Fc and an anti-APRIL antibody promote expression of pro-inflammatory mediators and inhibit phagocytosis and chemotaxis in the THP-1 monocyte cell line, suggesting that these effects are mediated by APRIL reverse signalling (Lee et al., 2010a). APRIL reverse signalling could not, however, be demonstrated in primary macrophages (Nys et al., 2013).

Several recent studies have reported that reverse signalling by members of the TNFSF is physiologically relevant for regulating axon and dendrite growth. TNFR1-activated TNF reverse signalling enhances the growth of sympathetic and sensory axons *in vitro* and plays a role in establishing target field innervation *in vivo* (Kisiswa et al., 2013, 2017; Wheeler et al., 2014). CD40-activated CD40L reverse signalling enhances sympathetic axon growth *in vitro* and promotes the sympathetic innervation of a subset of tissues *in vivo* (McWilliams et al., 2015). CD40L reverse signalling has also been recently shown to exert opposite effects *in vivo* on the growth and complexity of excitatory hippocampal pyramidal neuron dendrites and inhibitory striatal medium spiny neuron dendrites (Carriba and Davies, 2017).

We have shown that modulation of NGF-stimulated ERK activation plays a key role in mediating the effects of TWE-PRIL reverse signalling on axon growth. Likewise, modulation of ERK activation is a key step in effecting the influence of TNFR1-activated TNF reverse signalling on axon growth (Kisiswa et al., 2013). However, whereas TNF reverse signalling enhances NGF-stimulated ERK activation, TWE-PRIL reverse signalling appears to have the opposite effect. Activation of PKC is a key step in ERK activation by TNF reverse signalling, and PKC activation has been shown to mediate the effects of CD40L reverse signalling on dendrite growth in the CNS. However, different PKC isoforms have been implicated in mediating the promotion and inhibition of dendrite growth by CD40L reverse signalling in different CNS neurons (Carriba and Davies, 2017). Whether PKC and whether distinct PKC isoforms play a role in mediating the axon growth inhibitory effects of TWE-PRIL reverse signalling are interesting questions for future research as is the potential modulation of other signalling pathways that influence axon growth.

**Fig. 8.**
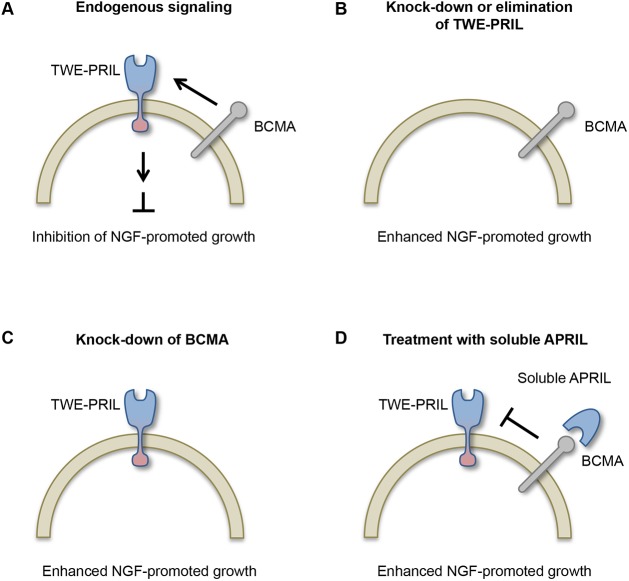
**Schematic outline of the suppression of NGF-promoted sympathetic axon growth by TWE-PRIL reverse signalling.** (A) Model illustrating suppression of NGF-promoted axon growth by BCMA-activated TWE-PRIL reverse signalling in developing SCG neurons (represented by the semi-circle). Arrows indicate the ERK-mediated intracellular signalling that links TWE-PRIL reverse signalling to axon growth. (B-D) Summary of the main experimental manipulations that underlie this model. (B) Selective knock down of TWE-PRIL by siRNA or the absence of TWE-PRIL in *April*^−/−^ mice removes the suppression signalling, resulting in enhanced NGF-promoted axon growth. (C) Knock down of BCMA by siRNA removes the activator of TWE-PRIL reverse signalling, resulting in removal of suppression signalling and enhanced NGF-promoted axon growth. (D) Soluble APRIL competes with endogenous TWE-PRIL for binding to BCMA, resulting in reduced or absent activation of TWE-PRIL reverse signalling, removal of suppression signalling and enhanced NGF-promoted axon growth.

In summary, our data suggest that TWE-PRIL reverse signalling is a physiologically relevant regulator of NGF-promoted sympathetic axon growth (Fig. 8). As TWE-PRIL reverse signalling has yet to be reported in any system, our study not only shows that TWE-PRIL can mediate reverse signalling, but also provides evidence for the first physiological role for TWE-PRIL.

## MATERIALS AND METHODS

### Animals

This study was conducted on tissues obtained from CD1 wild-type mice (*Mus musculus*) and mice with a null mutation in the *April* gene (a gift from Raif Geha, Boston Children's Hospital, Harvard Medical School, Cambridge, MA, USA) that were backcrossed into a CD1 background. The *April*-null mice lack half of exon 3 and exons 4 to 6 of the *April* gene, which encode 126 amino acids of the extracellular domain (Castigli et al., 2004). As such, these mice lack both APRIL and TWE-PRIL. Breeding, housing and genotyping was approved by the Cardiff University Ethical Review Board and was performed within the guidelines of the Home Office Animals (Scientific Procedures) Act, 1986.

### Neuron culture

SCG were dissected from embryonic and postnatal CD1 mice. The ganglia were trypsinized and plated at very low density (∼500 neurons per dish) in poly-ornithine/laminin-coated 35 mm or four-well tissue culture dishes (Greiner) in serum-free Hams F14 medium (Davies et al., 1993) supplemented with 0.25% Albumax I (Life Technologies).

Neuronal survival was estimated by counting the number of attached neurons within a 12×12 mm grid in the centre of each dish 2 h after plating and again after 24 h, and expressing the 24 h count as a percentage of the 2 h count. Analysis of the size and complexity of axon arbors was carried out by labelling the neurons at the end of the experiment with the fluorescent vital dye calcein-AM (Life Technologies). For every condition in each experiment, images of at least 50 neurons were digitally acquired by fluorescence microscopy and analysed to obtain total axon length, number of branch points and Sholl profiles (Gutierrez and Davies, 2007). Statistical analyses were performed using one way ANOVA with Bonferroni-Holm post hoc test. Pair-wise comparisons were made using Student's *t*-test.

For siRNA experiments, dissociated suspensions of SCG neurons were electroporated with Silencer Select siRNAs (Life Technologies) directed against TWE-PRIL/APRIL, APRIL alone, TWEAK alone, BCMA or TACI, or with a scrambled control siRNA. Co-transfection of a GFP expression plasmid allowed identification of the transfected neurons and imaging of their processes.

Recombinant NGF and APRIL were obtained from R&D Systems. Where indicated, the culture medium was supplemented with the caspase inhibitor Boc-D-FMK (Calbiochem) to prevent neuronal apoptosis in the absence of or in low concentrations of NGF. Expression plasmids were obtained from OriGene and the truncated TWE-PRIL construct was synthesized by Eurofins.

### Immunocytochemistry

The culture medium was gently aspirated and the cultures were washed with PBS at 37°C. Cells were fixed with either ice-cold methanol for 5 min or freshly made 4% paraformaldehyde (Sigma-Aldrich) in 0.12 M phosphate buffer (pH 7.2) for 12 min. The fixative was removed and after washing with PBS, the cultures were blocked for ∼1 h at room temperature in 5% BSA containing 0.2% Triton X-100 (Sigma-Aldrich). The cultures were then incubated overnight at 4°C with primary antibody in PBS containing 1% BSA (Sigma-Aldrich) and were gently agitated on an orbital shaker. After extensive washing in PBS, the cultures were incubated with fluorophore-conjugated secondary antibody (Alexa Fluor 488/546, Thermo) in 1% BSA for 1 h in the dark at room temperature. Following serial washes in PBS, the nuclei were counterstained with DAPI (1:10,000 dilution; Life Technologies) for 5 min. The cultures were imaged with either a Zeiss Axiovert 200 Inverted Fluorescence microscope or with a Zeiss LSM 710 Confocal Laser Scanning Microscope using Zen software. The following primary antibodies were used: rabbit polyclonal anti-APRIL (1:300, ab64967, Abcam), rabbit polyclonal anti-APRIL (1:300, 19976, Thermo), rat monoclonal anti-APRIL (1:300, ALX-804-141-C100, Enzo), rabbit polyclonal anti-BCMA (1:300, ab5972, Abcam), rabbit polyclonal anti-TWEAK (1:300, ab37170), rabbit polyclonal anti-TACI (1:200, ab79023) and mouse monoclonal anti-βIII tubulin (1:500, MAB1195, R&D Systems).

### Analysis of SCG fibres *in vivo*

Analysis of sympathetic fibre growth from the SCG was carried out by immunolabelling-enabled three-dimensional imaging of solvent-cleared organs (iDISCO) (Renier et al., 2014) of E16 *April*^+/+^ and *April*^−/−^ heads. Briefly, the heads were fixed in 4% paraformaldehyde for 24 h and serially dehydrated in a series of increasing methanol concentrations in water. The samples were then incubated overnight in 66% dichloromethane (DCM)/33% methanol, bleached in chilled 5% H_2_O_2_ to reduce tissue auto-fluorescence and then serially rehydrated in a series of decreasing methanol concentrations in water. After rehydration, the heads were washed in PBS containing 0.2% Triton X-100 (PTx.2) and were incubated in permeabilization solution (PTx.2, 20% DMSO and 0.3 M glycine) for 36 h at 37°C. After permeabilization, the samples were washed twice in PTx.2 and then incubated in blocking solution (6% donkey serum, 10% DMSO in PTx.2) for 36 h at 37°C. The heads were then incubated with rabbit polyclonal anti-tyrosine hydroxylase antibody (1:300, AB152, Millipore) in PTwH (0.2% Tween-20 and 10 mg/ml heparin in PBS) containing 5% DMSO and 3% donkey serum for 72 h at 37° C. Following extensive washing in PTwH, the samples were incubated with donkey anti-rabbit 647 Alexa Fluor secondary antibody (1:300, A-31573, Life Technologies) in PTwH plus 3% donkey serum for 72 h at 37°C. After further washing in PTwH, samples were cleared by dehydration in a series of methanol concentrations in water followed by 3 h incubation in 66% DCM/33% methanol. After washing in 100% DCM, the samples were placed in dibenzyl ether (DBE) until clear and imaged while submerged in DBE.

The specimens were imaged using a LaVision Light Sheet Ultramicroscope. Optical sections (5 µm) were acquired using a Neo high sensitivity CMOS camera. Captured *z*-axis images were analysed with Imaris 9 software (Bitplane Scientific Solutions), which allows for reconstruction of TH-positive fibres in three dimensions. The Filament Tracer module was used to detect fibres within the selected region of interest with semi-manual segmentation and the Surface Creation Module allowed for automized three-dimensional computerized reconstructions of TH-positive fibres after manual threshold optimization (Schindelin et al., 2012). The *April*^−/−^ data are expressed as a percentage of the mean of *April*^+/+^ data. All imaging and quantification was performed blind with genotypes determined after TH quantification.

### RT-qPCR

The levels of *April*, *Twe-pril*, *Tweak*, *Bcma*, *Taci*, TrkA and p75mRNAs were quantified by RT-qPCR in total RNA extracted from dissected SCG relative to a geometric mean of mRNAs encoding the housekeeping enzymes glyceraldehyde phosphate dehydrogenase (GAPDH) and succinate dehydrogenase (SDHA). Total RNA was extracted from dissected tissues with the RNeasy Mini Lipid extraction kit (Qiagen) and 5 µl was reverse transcribed for 1 h at 45°C using the AffinityScript kit (Agilent) in a 25 µl reaction according to the manufacturer's instructions. cDNA (2 µl) was amplified in a 20 µl reaction volume using Brilliant III ultrafast QPCR master mix reagents (Agilent). QPCR products were detected using dual-labelled (FAM/BHQ1) hybridization probes specific to each of the cDNAs (MWG/Eurofins). The PCR primers were: *April* forward, 5′**-**CTG TCC TTC CTA GAT AAT G-3′; *April* reverse, 5′**-**CTA GTG ACA CTC TGA CAC**-**3′; *Bcma* forward, 5′-TGA CCA GTT CAG TGA AAG G**-**3′; *Bcma* reverse, 5′**-**GGG TTC ATC TTC CTC AGC**-**3′; *Tweak* forward, 5′-CTT GCT CTT CTT TAA CAT CC-3′; *Tweak* reverse, 5′-GAT AAG TAG GGG CTT TGG-3′; *Twe-pril* forward, 5′-ATT CTC AGC CAC AGC AGC-3′; *Twe-pril* reverse, 5′-TTC GCC CCA TCC TTC CAG-3′; *Taci* forward, 5′-CTC AAG GAA ATC CTG TG-3′; *Taci* reverse, 5′-GAA TTT GCA GAA GTC TGT AC-3′; *TrkA* forward, 5′-CTG TGT CCA TCA CAT CAA-3′; *TrkA* reverse, 5′-GAA GGT TGT AGC ACT CAG-3′; *p75* forward, 5-ACC AGA GGG AGA GAA ACT-3′; *p75* reverse, 5′-GCA GGC TAC TGT AGA GGT-3′; *Gapdh* forward, 5′-GAG AAA CCT GCC AAG TAT G-3′; *Gapdh* reverse, 5′-GGA GTT GCT GTT GAA GTC-3′; *Sdha* forward, 5′-GGA ACA CTC CAA AAA CAG-3′; and *Sdha* reverse, 5′-CCA CAG CAT CAA ATT CAT-3′. Dual-labelled probes were: *April*, 5′-FAM-CAC CAA ATT CTC CTG AGG CT-BHQ1-3′; *Bcma*, 5′-FAM-CGT ACA CGG TGC TCT GGA TCT TCT T-BHQ1-3′; *Tweak*, 5′-FAM-CCA CCA CAA CTA TCC ACC TCA C-BHQ1-3′; *Taci*, 5′-FAM-CGC TGG CTC CTC TGG CTG-BHQ1-3′; *TrkA*, 5′-FAM-CGC CAG GAC ATC ATT CTC AAG T-BHQ1-3′; *p75*, 5′-FAM-ACA GCG ACA GCG GCA TCT-BHQ1-3′; *Gapdh*, 5′-FAM-AGA CAA CCT GGT CCT CAG TGT-BHQ1-3′; and *Sdha*, 5′-FAM-CCT GCG GCT TTC ACT TCT CT-BHQ1-3′. The dual-labelled *Twe-pril* probe (Eurogentec) was modified with locked nucleic acids (LNA) at the underlined positions and was; 5′-FAM-CCA GGA CAT CAG GAC TCT-BHQ1-3′. Forward and reverse primers were used at a concentration of 150 nM each and dual-labelled probes were used at a concentration of 300 nM. PCR was performed using the Mx3000P platform (Agilent) using the following conditions: 45 cycles of 95°C for 11 s and 60°C for 35 s. Standard curves were generated for each cDNA for every real-time PCR run, by using serial threefold dilutions of reverse transcribed adult mouse spleen total RNA (AMS Biotechnology). Three to six separate dissections were performed for each age.

### Immunoblotting

Whole SCG or SCG neurons cultured at high density in 96-well plates (>5000 cells/well) were lysed in ice-cold RIPA lysis buffer supplemented with protease and phosphatase inhibitor cocktail mix (Sigma-Aldrich). Insoluble debris was removed by centrifugation. Equal quantities of protein were run on pre-cast 4-20% SDS-PAGE gels (BioRad) and were transferred to PVDF membranes using a TransBlot Turbo Apparatus (BioRad), which were then incubated with blocking solution for 1 h at room temperature (5% non-fat dry milk in PBS with 0.1% Tween-20, PBS-T). After washing with PBS-T the blots were probed overnight at 4°C with the following antibodies: rabbit anti-TH (1:1000, AB152, Millipore), rabbit anti-phospho-ERK1/ERK2 (1:1000, ab9101, Cell Signaling Technologies), rabbit anti-total ERK1/ERK2 (1:1000, ab9102, Cell Signaling Technologies), rabbit anti-TrkA (1:500, ab2505, Cell Signaling Technologies) or mouse anti-β-III tubulin (1:5000, MAB1195, R&D). Bound primary antibodies were visualized with HRP-conjugated anti-mouse or anti-rabbit secondary antibodies (1:2000, W4021 or W4011, Promega) and Immunocruz Luminol reagent (Santa Cruz) and Amersham Hyperfilm ECL (GE Life Sciences). Densitometry was carried out using Image Studio Lite (Li-cor Biosytems). The levels of phospho-ERK1 and phospho-ERK2 were normalized to the levels of total ERK1 and ERK2.

## Supplementary Material

Supplementary information
