# Supplementary material for: TWE-PRIL reverse signalling suppresses sympathetic axon growth and tissue innervation
Source: Development. 2018 Nov 19;145(22):dev165936. doi: 10.1242/dev.165936 (PMC6262789; doi:10.1242/dev.165936)
Supplement: Supplementary information [file develop-145-165936-s1.pdf]

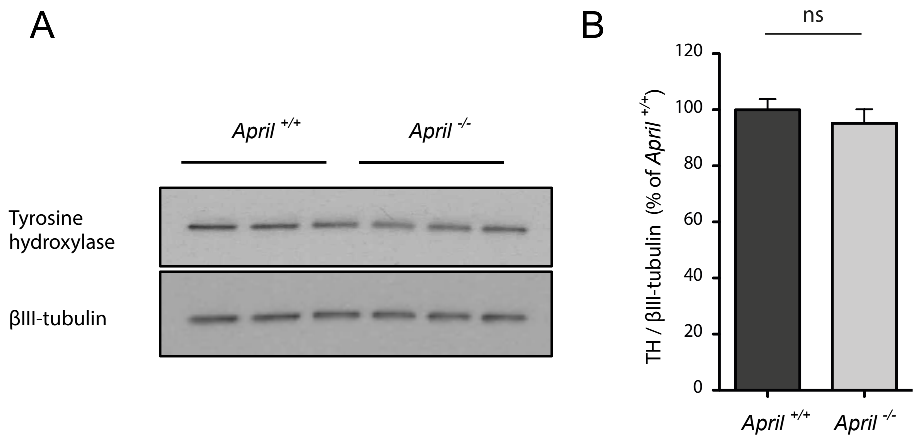

Figure S1. TH levels are not significantly different between the SCG of *April*$^{+/+}$ and *April*$^{-/-}$ mice. (**A**) Western blot of TH and βIII-tubulin in SCG dissected from 3 separate litters of *April*$^{+/+}$ and 3 separate litters of *April*$^{-/-}$ newborn mice (6 ganglia per litter). (**B**) Quantification by densitometry of the ratio of TH to βIII-tubulin levels in the Western blots shown in A normalized to the ratio in *April*$^{+/+}$ mice.

Development • Supplementary information

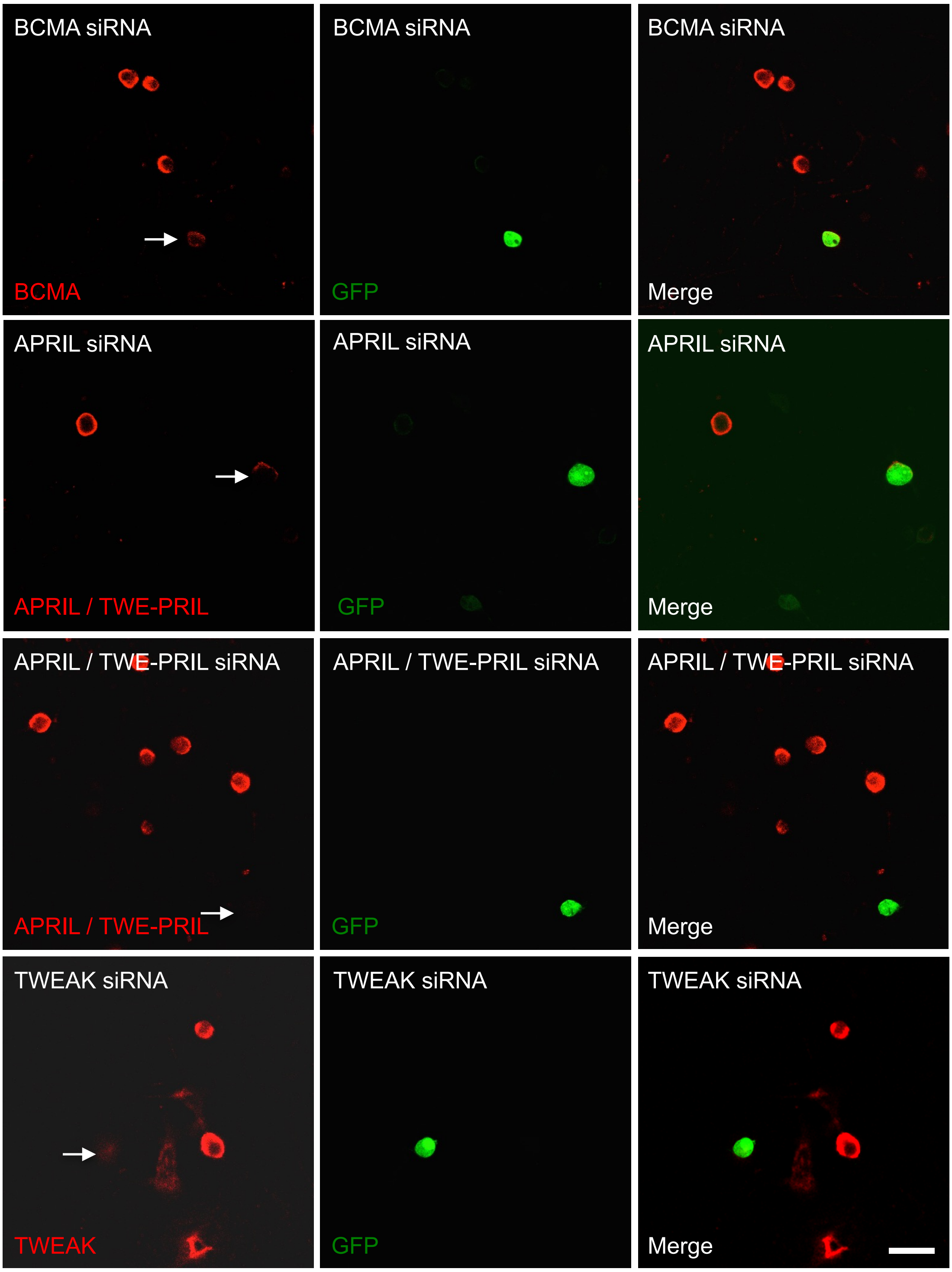

Figure S2. siRNAs eliminate immunoreactivity for the proteins to which they are directed. Representative images of P0 SCG neurons that were co-transfected with a GFP expression plasmid to indicate which neurons were transfected (green and arrows) and the siRNAs indicated. The neurons were subsequently stained for expression of the indicated proteins (red) 16 h after incubation in medium containing 1 ng/ml NGF and 25 µM Boc-D-FMK. Scale bar, 100 µm.

Development • Supplementary information

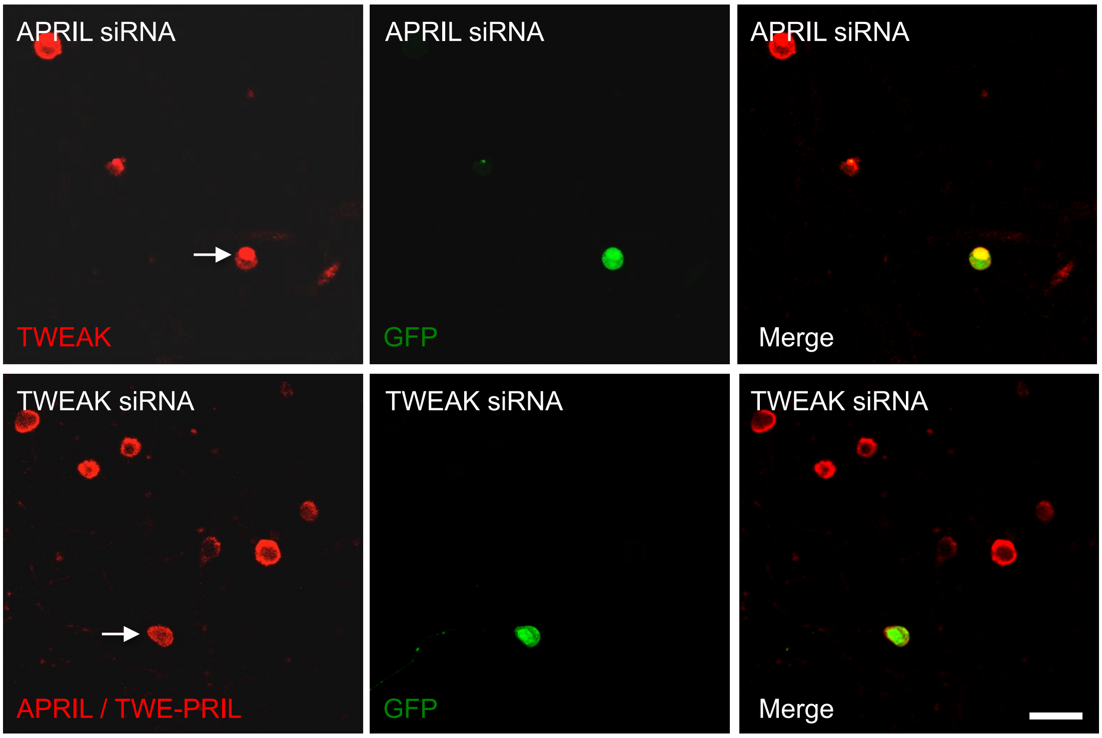

Figure S3. Specificity of siRNA knockdown. Representative images of P0 SCG neurons that were co-transfected with a GFP expression plasmid to indicate which neurons were transfected (green and arrows) and the siRNAs indicated. The neurons were subsequently stained for expression of the indicated proteins (red) 16 h after incubation in medium containing 1 ng/ml NGF and 25 μM Boc-D-FMK. Scale bar, 100 μm.

Development • Supplementary information

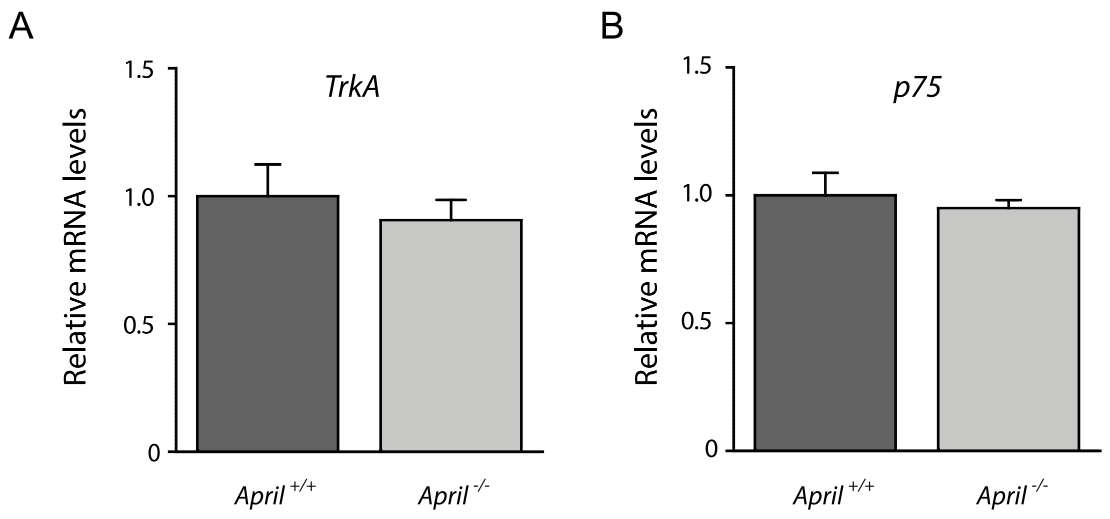

Figure S4. Deletion of *April* does not significantly affect expression of mRNAs encoding NGF receptors in SCG. Bar charts of the levels of *TrkA* mRNA (**A**) and *p75* mRNA (**B**) relative to the geometric mean of *Gapdh* and *Sdha* mRNA in SCG dissected from 4 separate litters of *April*$^{+/+}$ and 4 separate litters of *April*$^{-/-}$ mice at 16 days gestation (6 or more ganglia per litter).
